# Structured training on gross motor skills and physical fitness in 4–5-year-old children

**DOI:** 10.3389/fped.2024.1466911

**Published:** 2024-12-13

**Authors:** Sheng Quan, Yutong Liao, Yulin Ji, Shuwen Zheng

**Affiliations:** ^1^ Department of Police Tactics, Fujian Police College, Fuzhou, Fujian, China; ^2^ College of Education, Fujian Normal University, Fuzhou, Fujian, China

**Keywords:** structured training, young children, gross motor, physical fitness, intervention

## Abstract

**Objective:**

Preschool children are in a period of rapid physical development, and improving their gross motor skills and physical fitness is quite important for their health. This study aimed to investigate the effectiveness of a structured physical training program in improving Chinese preschool children's gross motor development and physical fitness.

**Method:**

A sample of 80 children aged 4 to 5 from Fujian, China, were randomly assigned to the intervention group (*N* = 41), which received a 15-week structured physical training, while the control group (*N* = 39) continued with their daily physical activity. The Test of Gross Motor Development-3, and the National Physical Fitness Measurement Standards Manual -Preschool Children Version (2003) were assessed before and after the intervention.

**Results:**

A series of ANCOVA analyses revealed significant group differences in aspects of gross motor skills (*F* = 10.17, *p* < 0.01) including locomotor skills (*F* = 5.31, *p* < 0.05) and ball skills (*F* = 15.09, *p* < 0.001) after controlling the effect of the age, sex, and pre-test scores. Moreover, the results also indicated a higher improvement in young children's physical fitness (*F* = 91.33, *p* < 0.001) including their body shape (*F* = 5.05, *p* < 0.05), health-related fitness (*F* = 43.09, *p* < 0.001), and skill-related fitness (*F* = 61.47, *p* < 0.001) in the intervention group over the control group. The results demonstrated that the effect size of the structured training on young children's health-related fitness (*η*^2^ = 0.38) and skill-related fitness (*η*^2^ = 0.50) was much stronger than on children's body shape (*η*^2^ = 0.07).

**Conclusion:**

The structured training program effectively improved young children's gross motor skills and physical fitness.

## 1 Introduction

Young children's physical activity levels have decreased dramatically over recent decades, which has significant public health implications and has evolved into a global public issue (1, 2). The 2030 Agenda for Sustainable Development proposed “Health and Wellbeing” as a goal to maintain health while simultaneously promoting individual well-being (3). Physical activity is related to children's motor skills and physical fitness which contribute to their long-term participation in sports and keeping healthy (4–6). As a populous country, China has always prioritized the improvement of young children's health, recognizing its importance in enhancing national health. Previous studies have shown alarming rates of obesity and overweight among Chinese young children (7, 8). Focusing on physical activity and motor development is beneficial for Chinese young children to maintain a healthy life.

Motor development, also known as “perceptual-motor development”, involves the intricate interaction of the brain, nervous system, and muscles, enabling children to manipulate objects and explore their surroundings (9). Motor development associated with both cognitive and social outcomes plays an important role in one's long-term development (10–12). Gross motor development can be described as the obtaining of control and use of the large muscles in the body. It is widely recognized that early childhood is a period of rapid gross motor development (13, 14). During preschool years, children develop and acquire basic gross motor skills which include locomotor skills such as jumping, running, skipping, galloping, hopping, and manipulative skills such as ball handling skills [eg., (15, 16)].

Physical fitness refers to the overall performance of young children's physical functions during physical activity (5). Moreover, physical fitness can be divided into two broad categories: health-related fitness and skill-related fitness. Specifically, health-related fitness includes anthropometric parameters, muscular strength, flexibility, etc. Skill-related fitness includes agility, power, balance, etc. (1, 17). Studies have demonstrated that low levels of physical fitness are associated with young children's health problems and high levels of physical fitness can protect young children from obesity and metabolic diseases (18–20). These findings highlight the importance of promoting physical fitness among young children.

Physical activity is regarded as one of the important factors in promoting gross motor skills and physical fitness. Studies have indicated that compared to free play, school-based physical activity is more effective in improving gross motor skills and physical fitness for young children (21). Structured training is a kind of physical activity that is characterized by clear instructions and continuous feedback, focusing on immediate correction and bringing greater satisfaction and ownership (22). Additionally, Structured training emphasizes on the effectiveness of movement and promotes the development of basic motor skills, and physical fitness linearly by considering a progression of skills from simple to complex (23).

While numerous studies have identified the critical role of physical activity in promoting children's gross motor skills (24, 25) and physical fitness (26, 27), limited research has focused on the effect of structured training on Chinese young children. Therefore, we designed an intervention study to examine the effectiveness of a structured preschool training program in improving young children's gross motor skills and physical fitness. This study aims to fill the gap in the literature and provide valuable insights into early childhood education practices in China.

## 2 Material and methods

### 2.1 Participants

**Figure 1:**
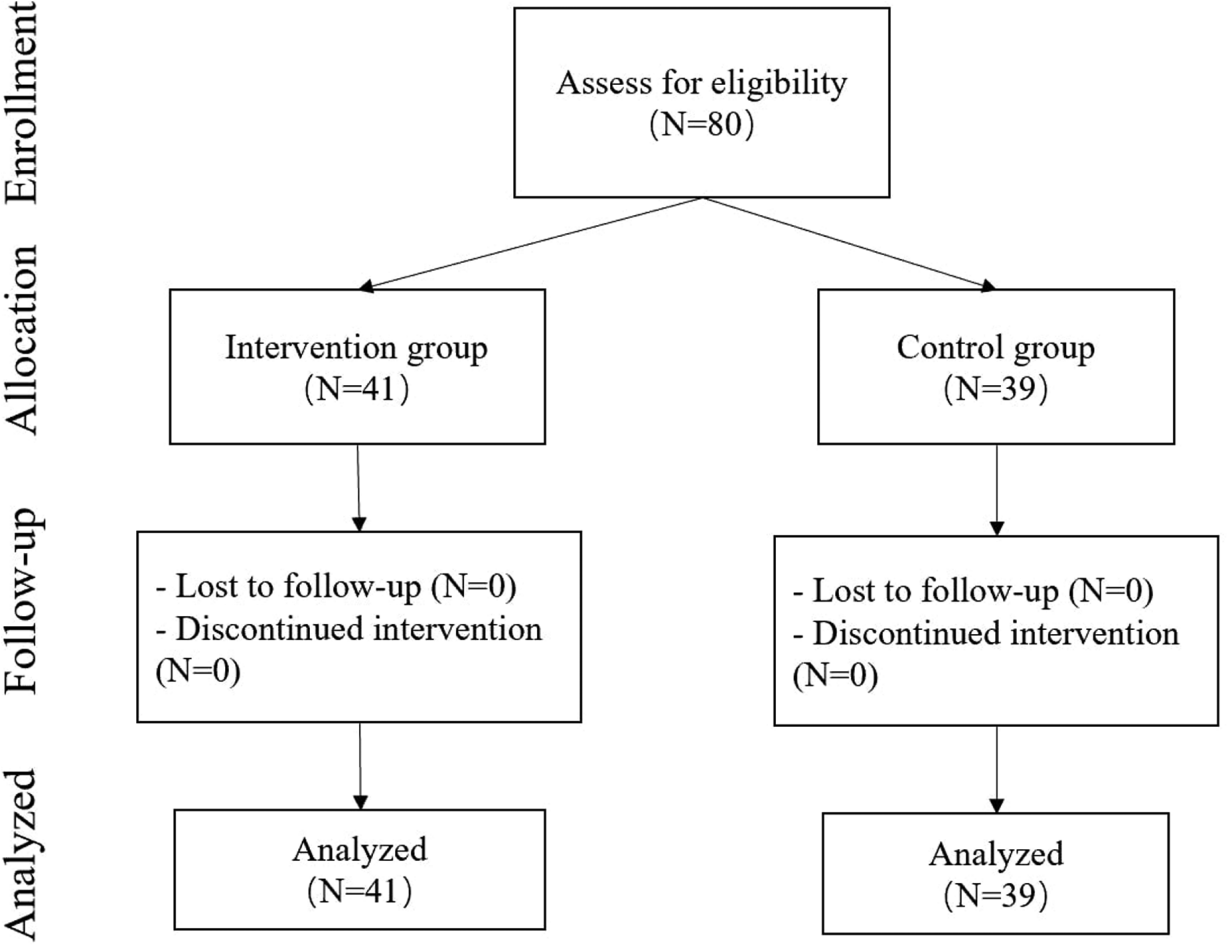
CONSORT flow diagram.

This research was conducted in Fuzhou, the capital of Fujian province in southeast China, which has a population of over 10 million people and a rapidly rising economy. As shown in Figure 1, the participants consisted of 80 children (42 boys and 38 girls) aged 4 to 5 years (in the intervention group, 41 children with 21 boys and 20 girls, M_age_ = 4.50 SD = 0.38; in the control group, 39 children with 21 boys and 18 girls, M_age_ = 4.48 SD = 0.42). This study enrolled participants in two classes at two public preschools with good standards in Fuzhou city where the majority of the children came from middle-class families and above. The inclusion criteria were having a minimum class attendance of 80% and having no pathological condition that would hinder physical activity. Participants from the two classes were then divided into two groups. Participants in both groups engaged in similar activity levels about 1 h per day. The demographic characteristics of the participants are shown in Table 1. The local Ethics Committee approved the study, and informed consent was obtained from the participants' parents.

**Table 1:** Characteristics of preschool children.

	Control group (*N* = 39)	Intervention group (*N* = 41)	*p*
Age (year)	4.48 (±0.42)	4.50 (±0.38)	0.786
Height (m)	1.08 (±0.49)	1.08 (±0.48)	0.953
Weight (kg)	19.03 (±2.62)	19.41 (±2.22)	0.484
BMI	17.43 (±1.76)	17.87 (±1.44)	0.753

Scores are presented as mean (±SD).

### 2.2 Measures

#### 2.2.1 Test of gross motor development 3nd edition (TGMD-3)

Gross motor skills were assessed using the Test for Gross Motor Development-3rd Edition (TGMD-3) (15, 28). The TGMD-3 is a norm- and criterion-referenced measure designed for children aged 3 to 10. TGMD-3 includes locomotor and ball skills domains for a total of 13 fundamental movement skills, with the locomotor domain containing six skill tests and the ball skills domain containing 7 tests. Each task includes several behavioral components that are expressed as performance criteria, and each task was scored based on the performance, with a score of one point for correct execution and zero points for not performing the standard. The scores for the two domains could be added together to get a total gross motor score.

#### 2.2.2 The national physical fitness measurement standards manual -preschool children version (NPFM-PC, 2003)

The government of China published the NPFM-PC in 2003. This measure comprises anthropometric values and physical fitness tasks (29). The anthropometric values measure children's body shape scored by height and weight. Young children's health-related fitness is assessed using the tennis throwing (muscular strength), and the sit-and-reaching test (flexibility). Meanwhile, young children's skill-related fitness is evaluated through the 10 m shuttle running, standing long jump, double-leg timed hopping and balance beam walking. Each task is scored by several performance criteria with scores ranging from 1 to 5. Participants performed two trials for each task with at least one minute of rest between trials, and the same researcher conducted all the tests.

### 2.3 Design and intervention

**Figure 2:**
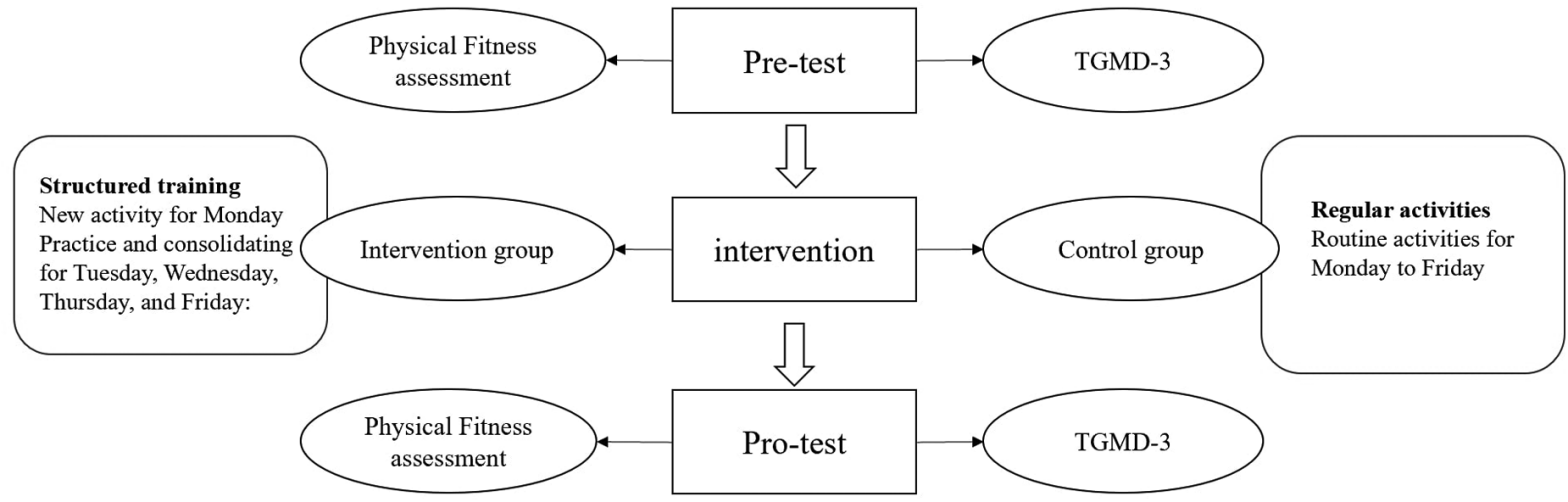
Intervention design procedures.

As shown in Figure 2, the intervention group participated in a structured training program designed to promote young children's gross motor skills and physical fitness. Firstly, by analyzing the physical development characteristics of young children and combining them with the movement structure analysis, we identified the levels of gross motor skills and physical fitness that 4–5-year-olds were expected to achieve. Secondly, based on the constraints-led approach developed by Newell (30), the intervention group promoted children's motor development by changing the environment (situation, venue, equipment) and tasks. Finally, the intervention was modified and adjusted according to the training process. Table 2 presents the main activities in the structured training program. The program centered on 3 types of movements (mobility movements, stability movements, and object control) focusing on gross motor development and physical fitness. The gross motor training content included walking, running, jumping, crawling, hitting, throwing, and catching. The physical fitness training contained balance, muscular strength, flexibility, and agility. Additionally, mini-games were used to interest young children in practice. The intervention lasted for one semester about 16 weeks. The control group participated in the traditional “walking, running, jumping, throwing, climbing, drilling, and crawling” as the main content of the physical activity.

**Table 2:** Description of the structure training program.

Month	Main activity
September	Locomotor Skills: Marching, walking in single file, tiptoe walking, walking backward, walking with eyes closed
	Manipulative Skills: Rolling a ball,dribbling, self-tossing and catching
	Stability Skills: Balancing with equipment, “swallow” balance
October	Locomotor Skills: Running on elbows and knees, crawling on hands and feet, belly crawling, back crawling
	Manipulative Skills: Passing a ball, catching a ball, dribbling
	Stability Skills: Straight body side roll, forward roll
November	Locomotor Skills: Straight-line running, chase running, dodging, curved running, shuttle running, high knee running
	Manipulative Skills: Hitting a stationary ball with a racket (forehand and backhand), throwing for distance, throwing for accuracy
	Stability Skills: Tucked body side roll, backward roll
December	Locomotor Skills: Vertical jump, hopping with both feet, single-leg hopping, running jump, vaulting
	Manipulative Skills: Hitting a ball with a racket (forehand and backhand), kicking a ball
	Stability Skills: Support swing, hanging swing

The present study employed a quasi-experimental design with a quantitative approach. The intervention and control groups were consistent in the frequency and duration of the physical activity. In addition, the intervention and control groups engaged in the same activity levels per day. The interventions were conducted by preschool teachers. Researchers provided intervention training for teachers every two weeks. The intervention lasted for 16 weeks with 25 min activities conducted 5 times a week. Specifically, the intervention on Monday focused on teaching the new movement skills, and the other four interventions in a week were outdoor activities for practicing and consolidating, which took place on Tuesday, Wednesday, Thursday, and Friday respectively. Participants in both the intervention group and control group completed pre- and post-intervention assessments including TGMD-3 and NPFM-PC. The assessments were conducted by two trained researchers on a flat and obstacle-free space at the preschool outdoor playground, and the TGMD-3 and NPFM-PC assessments were completed in one week.

### 2.4 Statistics

IBM SPSS Statistics 23.0 was used for the statistical analysis. All results are presented as mean ± standard deviation A Shapiro-Wilk test was conducted to confirm data normality. A series of ANCOVA analyses were used to investigate whether group differences between the service-learning group and the control group existed while accounting for potential preexisting differences. This method allows for group comparison of the post-scores while considering the potential influence of differences in the pre-scores. In the ANCOVA analyses, child age, sex, and pre-scores of TGMD-3 and NPFM-PC were entered as covariates, and post-scores were entered as the dependent variables. Gross motor development and physical fitness scores were obtained using calculation methods in the literature. *P* value < 0.05 was considered statistically significant.

## 3 Results

### 3.1 Pre and post-test results of descriptive information

Thirty-nine young children in the intervention group and forty-one young children in the control group took the TGMD-3 and NPFM-PC for the pre-test and the post-test in this study. The data was analyzed statistically. The descriptive analysis of the TGMD-3 was shown in Table 3 and the descriptive analysis of the NPFM-PC was presented in Table 4. The sample included 3 boys and 32 girls in the intervention group, and 11 boys and 24 girls in the control group.

**Table 3:** Pre and post-test results of descriptive information on gross motor development.

	Control group (*N* = 39)		Intervention group (*N* = 41)	
	Pre-test	Post-test	Pre-test	Pro-test
Locomotor skills	3.75 (±1.58)	13.93 (±1.58)	11.95 (±3.33)	18.81 (±2.43)
Run	0.90 (±0.30)	2.44 (±0.74)	1.95 (±0.86)	3.03 (±0.44)
Gallop	0.25 (±0.44)	2.21 (±0.65)	2.56 (±1.12)	3.49 (±0.82)
Hop	0.80 (±0.41)	2.85 (±0.53)	1.23 (±1.01)	2.92 (±0.96)
Skip	0.47 (±0.51)	1.80 (±0.51)	0.87 (±0.81)	2.15 (±1.14)
Jump	0.88 (±0.34)	2.44 (±0.63)	2.00 (±0.80)	3.03 (±0.60)
Slide	0.45 (±0.50)	2.17 (±0.59)	3.33 (±1.22)	3.92 (±0.27)
Ball skills	2.12 (±1.32)	9.88 (±1.93)	9.28 (±3.13)	16.36 (±2.97)
Strike	0.10 (±0.30)	0.00 (±0.00)	1.38 (±0.75)	2.03 (±0.74)
One-hand strike	0.05 (±0.22)	0.00 (±0.00)	0.00 (±0.00)	0.49 (±0.51)
Dribble	0.95 (±0.22)	2.29 (±0.64)	1.95 (±1.03)	3.33 (±0.98)
Catch	0.30 (±0.46)	1.66 (±0.66)	1.77 (±0.63)	2.28 (±0.60)
Kick	0.63 (±0.49)	1.88 (±0.75)	1.95 (±0.86)	2.69 (±0.57)
Overhand throw	0.00 (±0.00)	2.00 (±0.71)	1.03 (±0.49)	3.03 (±0.84)
Underhand throw	1.21 (±0.66)	2.51 (±0.76)	1.21 (±0.66)	2.51 (±0.76)
Overall gross motor	5.78 (±2.36)	23.80 (±2.97)	19.85 (±4.70)	33.32 (±3.79)

Scores are presented as mean (±SD).

**Table 4:** Pre and post-test results of descriptive information on physical fitness.

	Control group (*N* = 39)		Intervention group (*N* = 41)	
	Pre-test	Post-test	Pre-test	Pro-test
Body shape	4.17 (±1.43)	4.41 (±1.11)	4.07 (±1.44)	4.54 (±1.07)
Health-related fitness	2.93 (±1.46)	4.15 (±1.25)	3.79 (±1.69)	6.17 (±1.32)
Tennis throwing	2.83 (±1.45)	2.54 (±0.67)	1.89 (±0.99)	3.43 (±0.88)
Sit-and-reaching	0.10 (±0.44)	1.62 (±1.18)	1.90 (±1.09)	2.74 (±1.12)
Skill-related fitness	6.97 (±2.63)	8.64 (±2.12)	8.21 (±2.65)	13.43 (±2.69)
Standing long jumping	0.76 (±0.70)	2.32 (±1.58)	2.13 (±1.08)	3.49 (±1.58)
Balance beam walking	2.65 (±1.61)	2.29 (±0.51)	2.82 (±1.02)	3.97 (±0.71)
10-m shuttle running	1.56 (±0.78)	1.87 (±0.92)	1.82 (±0.51)	2.97 (±0.92)
Double-leg timed hopping	1.76 (±1.04)	2.07 (±0.57)	1.44 (±1.29)	3.75 (±1.58)
Overall Physical fitness	14.16 (±3.96)	17.18 (±3.00)	16.08 (±4.12)	24.09 (±3.77)

Scores are presented as mean (±SD).

### 3.2 The comparison of gross motor and physical fitness

According to the covariance analysis results (ANCOVA) presented in Table 5, after controlling for the effect of confounders (age, sex, pre-test scores), there was a significant difference between the intervention and control groups (*F* = 10.17, *p* < 0.01) in total gross motor scores. The effect size for this significant difference was calculated as 0.12 eta-square. Additionally, the two sub-scales were also compared. Results indicated that there was also a significant difference between the intervention and control groups (*F* = 5.31, *p* < 0.05) in locomotor skills. The effect size for this significant difference was calculated as 0.07 eta-square. Besides that, there was a statistically significant difference in ball skills between the two groups (*F* = 15.09, *p* < 0.001). The effect size for this significant difference was calculated as eta-squared 0.17.

**Table 5:** ANCOVA results statistics.

Variable	Source	*df*	*MS*	*F*	*P*	Ƞ^2^
Gross motor	Pre-test	1	149.92	15.12**	0.000	0.17
	Age	1	4.06	0.41	0.524	0.01
	Sex	1	46.78	4.72*	0.033	0.06
	Group	1	100.81	10.17**	0.002	0.12
Locomotor	Pre-test	1	43.60	12.18**	0.001	0.15
	Age	1	0.05	0.01	0.907	0.00
	Sex	1	0.47	0.13	0.719	0.00
	Group	1	19.00	5.31*	0.024	0.07
Ball skills	Pre-test	1	53.74	10.02**	0.002	0.12
	Age	1	0.50	0.09	0.763	0.00
	Sex	1	26.27	4.90*	0.030	0.06
	Group	1	80.92	15.09***	0.000	0.17
Overall Physical fitness	Pre-test	1	172.63	31.69***	0.000	0.30
	Age	1	2.17	0.40	0.530	0.01
	Sex	1	15.75	2.89	0.094	0.05
	Group	1	497.42	91.33***	0.000	0.60
Body shape	Pre-test	1	200.84	680.23***	0.000	0.90
	Age	1	0.00	0.01	0.931	0.00
	Sex	1	0.17	0.56	0.456	0.01
	Group	1	1.49	5.05*	0.028	0.07
Health-related fitness	Pre-test	1	14.11	10.38**	0.002	0.13
	Age	1	0.11	0.08	0.777	0.00
	Sex	1	11.99	8.82**	0.004	0.11
	Group	1	58.55	43.09***	0.000	0.38
Skill-related fitness	Pre-test	1	91.32	22.02***	0.000	0.26
	Age	1	0.50	0.12	0.729	0.00
	Sex	1	1.563	0.38	0.542	0.01
	Group	1	254.87	61.47***	0.000	0.50

* *p* < 0.05.

** *p* < 0.01.

*** *p* < 0.001.

As shown in Table 5, after controlling for the effect of confounders (age, sex, pre-test scores), the covariance analysis results (ANCOVA) indicated that there was a significant difference between the two groups (*F* = 91.33, *p* < 0.001) in total physical fitness scores. The effect size for this significant difference was calculated as 0.60 eta-square. In addition, there was also a significant difference between the intervention and control groups in body shape (*F* = 5.05, *p* < 0.05), health-related fitness (*F* = 43.09, *p* < 0.001), and skill-related fitness (*F* = 61.47, *p* < 0.001) after controlling the effect of the age, sex, and pre-test scores. The effect size for this significant difference was calculated as 0.07 eta-square, 0.38 eta-square, and 0.50 eta-square respectively.

## 4 Discussion

The present study explored the potential gross motor development and physical fitness benefits of the preschool-based physical activity program. Data was collected with children in an intervention group and a control group (children who did not engage in the current program) at both the start and the end of the intervention. ANCOVA analyses were used to compare the post-scores of two groups while considering the possible effect of pre-existing differences by using the pre-scores as a covariate in the analyses. The findings of this study underscore the significant impact of structured physical activity on the gross motor development and physical fitness of young children. These results align with and extend previous research in several important ways. However, this study adds to the existing literature by specifically focusing on Chinese preschool children, a demographic that has been underrepresented in prior research.

The results revealed that structured physical activity could improve young children's gross motor development including locomotor skills and ball skills. The structured physical activity of this study included different types of movement practices that effectively promote young children's comprehensive gross motor skills. The results of this study were consistent with previous findings which indicated that structured activity programs could benefit one's movement outcomes (24, 25). There exists a common misconception that young children are naturally active. However, children who are not instructed to engage in physical activities may develop their motor competency more slowly (31–33).

The findings of this study demonstrated that physical activity is critical for improving gross motor development during the preschool period. The preschool years are characterized by the appearance and mastering of various gross motor skills (14). Typically, children first develop or obtain the foundational mechanisms required for the development of motor skills. Then, children achieve the so-called motor development milestones, which are followed by the development of basic gross motor skills. These competencies subsequently manifest in a range of specialized movement abilities characteristic of older children and young adults (34).

This study also found that structured physical activity improved young children's physical fitness outcomes including health-related fitness, skill-related fitness, and their body shape. Previous research supports the results revealed in this study, demonstrating that structured school-based intervention had a positive effect on children's and adolescents' physical fitness (21, 26, 27). Specifically, a recent study by Lee et al. (35) indicated that school-based physical activity intervention had a positive influence on the health-related physical fitness of adolescents. In terms of skill-related fitness, a study by Wick (36) indicated that a 10-week strength-dominated exercise program improved young children's jump performance. As far as anthropometry is concerned, a study by Mo-Suwan et al. (37) suggested that a preschool-based physical program could prevent BMI growth in girls and may reduce obesity in young children. Structured physical activities usually have clear aims and systematic plans, which gradually increase intensity and complexity to provide comprehensive exercise for children's physical function. At the same time, structured training can help improve children's physical fitness in various aspects such as strength, flexibility, and coordination (22, 38).

The results also revealed that the effect size of structured physical activity on health-related fitness and skill-related fitness was stronger than on children's body shape. There are still arguments in the field of physical activity programs on lean and obese children's anthropometric characteristics. The results of this study support the findings of Dobbins et al. (27) which highlighted the influence of such programs on children's anthropometric improvements. However, the study of Thivel et al. (39) found that although school-based physical activity interventions in primary school children contribute to effective outcomes in terms of aerobic and anaerobic physical fitness, the result of the intervention was insignificant in inducing obesity in young children. There existed a significantly inverse relationship between anthropometric values and health-related physical fitness in children (40). Therefore, an effective structured preschool physical activity could improve young children's health-related physical fitness while reducing lean and obesity. However, dietary habits should not be ignored when it comes to improving young children's body shape (41, 42). This may explain why the effect size of preschool physical activity interventions on improving children's health-related and skill-related fitness was stronger than their effect on body composition in this study, especially if previous dietary habits were maintained.

The study's findings also have important implications for early childhood education policies and practices. As highlighted by Tortella et al. (43), there has been a growing recognition of the need to integrate structured physical activity programs into preschool curricula. The current study provides further evidence to support such initiatives, particularly in the Chinese educational context, where the government prioritizes improving the health of young children as part of its national health agenda (7, 8). Furthermore, the study supports the 2030 Agenda for Sustainable Development's goal of promoting health and well-being (3). By fostering early motor skill development and physical fitness, structured physical activity programs can contribute to the long-term health and well-being of individuals, ultimately benefiting society as a whole.

## 5 Limitations and future research directions

This study contributes valuable insights to the growing body of literature on structured physical activity interventions for preschool children. It underscores the potential of such programs to enhance gross motor skills and physical fitness, providing important implications for early childhood education and public health policy. However, this study has several limitations that should be addressed in future research. First, this study only included 4–5-year-old preschoolers, which may not be generalizable to all preschool age groups. Future research should consider including a broader age range to determine if the findings are consistent across different developmental stages. Besides that, this study focused on the effects of structural training on children's physical development. Future research could further focus on the effects of structure training on young children's attitudes and motivation as well as their cognitive and social development. Moreover, parental surveys were also necessary for future studies to deeply understand children's exercise habits for comprehensive analysis.

Second, although we used ANCOVA analyses to control the effect of the pre-test results on the intervention effect, we had to note that the baseline levels of gross motor development and physical fitness between the intervention groups and control groups were not balanced. If the pre-test is lower, the post-test level after the interventions will be much higher. Future studies should aim to ensure more balanced baseline characteristics between groups to strengthen the validity of the findings.

Third, this study used a quasi-experimental design, and relevant variables were not tightly controlled. The positive effect of the intervention may also be attributed to more complex factors. Future research should consider employing a randomized controlled trial (RCT) design to better isolate the effects of the structured training program and control for potential confounding variables.

Last but not least, the intervention duration was limited to 15 weeks. Longitudinal studies with extended follow-up periods are needed to assess the long-term sustainability of the improvements in gross motor skills and physical fitness observed in this study. This would provide valuable insights into the lasting impact of structured physical activity programs on young children's development.

## 6 Conclusion

In conclusion, the results found that structure training had a significant impact on aspects of gross motor skills including locomotor skills and ball skills. In addition, the results also indicated a higher improvement in young children's physical fitness including their body shape, health-related fitness, and skill-related fitness through structure training. Therefore, we suggest that structure training should be emphasized in preschool physical education to develop children's gross motor skills and physical fitness. Moreover, the results revealed that the effect size of the structured training on young children's health-related fitness and skill-related fitness was much stronger than on children's body shape. Given the effect of structure training on children's body shape is influenced by dietary habits, families and preschools should collaborate to improve young children's body shape.

## Data Availability

The raw data supporting the conclusions of this article will be made available by the authors, without undue reservation.
